# Potassium-Enriched Salt to Lower Stroke Risk: A #NephJC Editorial on the SSaSS Study

**DOI:** 10.1016/j.xkme.2022.100489

**Published:** 2022-05-25

**Authors:** Yoshinosuke Shimamura, Michael Turk, Md Abdul Qader, Shweta Shah, Joel M. Topf, Swapnil Hiremath

**Affiliations:** 1 Department of Nephrology, Teine Keijinkai Medical Center, Sapporo, Hokkaido, Japan; 2 Department of Medicine, Allegheny Health Network, Pittsburgh, PA; 3 Department of Pediatric Nephrology, Square Hospitals Ltd, Dhaka, Bangladesh; 4 Renal Section, Texas Children’s Hospital and Baylor College of Medicine, Houston, TX; 5 Department of Medicine, Oakland University William Beaumont School of Medicine, Auburn Hills, MI; 6 Department of Medicine, University of Ottawa, Ottawa, ON, Canada

**Keywords:** Hypertension, #NephJC, potassium, sodium, stroke, Twitter journal club

*#* *NephJC is a recurring twitter-based journal club. #NephJC editorials highlight the discussed article and summarize key points from the NephJC TweetChat.*

## Background

The prevalence of hypertension has increased substantially over time, even after adjusting for age, and so have the associated disabilities and deaths.^1^ Based on physiology principles and epidemiological studies, high sodium intake has been recognized as a key contributor to high blood pressure (BP). Previous randomized controlled trials (RCTs) have demonstrated that lowering sodium intake to <2 g/d versus maintaining sodium levels at ≥2 g/d reduces BP by about 3.5/1.8 mm Hg.^2^ Another meta-analysis showed that for every 50 mmol (∼1.15 g of sodium) decrease in 24-hour sodium excretion, the BP was lowered by 1.1/0.3 mm Hg.^3^ However, whether reducing sodium intake decreases adverse clinical outcomes is uncertain because RCTs have not had adequate power or longer-term follow-up to assess the effects on morbidity and mortality.

Another important issue in the landscape of salt in hypertension is the effect of potassium intake on BP. Potassium lowers BP via multiple mechanisms, by acting directly on the vasculature and by triggering a signaling cascade mediated by With-no-lysine kinases that activates a “switch” to reduce sodium absorption.^4^ Furthermore, a meta-analysis of 21 RCTs reported that increasing potassium intake lowers BP (∼3.5/2.0 mm Hg decrease) and the risk of stroke.^5^ Notably, although there was no gradient for the effect of achieved potassium intake (which was ∼30-60 mmol/d higher) and BP, there was an interaction with sodium intake.^5^ From the Dietary Approach to Stop Hypertension-Sodium trial, it was noted that increasing potassium intake was more effective at reducing BP with high sodium intake (5.9/2.9 mm Hg reduction) than with low sodium intake (2.2/1.0 mm Hg reduction).^6^

Although both sodium reduction and potassium supplementation have been studied in RCTs, the feasibility of implementation of these interventions has not been well established. In most trials of sodium reduction, including the Dietary Approach to Stop Hypertension-Sodium trial, the participants received packaged meals, thus representing feeding trials that are not feasible for routine practice.^6^, ^7^, ^8^ Additionally, the source and amount of sodium intake consumed vary across the world. A systematic review reported that Americans consume 3,400 mg (147 mEq) per day, whereas the residents of Northern China consume >11,000 mg (480 mEq) per day.^9^^,^^10^ Importantly, in China, that sodium intake is dominated by the salt added while cooking in the household, whereas it is dominated by manufactured foods (eg, cereals and baked foods) in North America.^9^

Salt substitutes, in which some of the sodium is replaced by potassium, represent an opportunity for implementing a low sodium and high potassium intervention. These substitutes have been demonstrated to lower BP but have not been powered to determine the effect on clinical outcomes.^11^, ^12^, ^13^ The Salt Substitute and Stroke Study randomized high-risk people to table salt (100% sodium chloride) or a salt substitute (a composition of 75% sodium chloride and 25% potassium chloride), with the primary outcome being the rates of stroke.^14^

## The Trial

The Salt Substitute and Stroke Study was a prospective, open-label, cluster RCT that comprised 600 villages in rural China. Sixty villages in each of 10 counties from 5 provinces were selected to recruit approximately 35 persons from each village and follow them for 5 years. The intervention group was provided the salt substitute, whereas the control group was advised to continue using regular salt. In this cluster RCT, the entire village was randomized to the salt substitute or usual care, thus obviating the issue of potentially randomizing members of the same family to different arms, as well as making it a pragmatic design for implementation. The study included people who had previously had a stroke, were aged greater than 60 years, and had poorly controlled hypertension. Poorly controlled hypertension was defined as a systolic BP of ≥140 mm Hg if on antihypertensive medication or ≥160 mm Hg if not on antihypertensives. People who used potassium-sparing diuretics, had “serious kidney disease,” were unlikely to live for more than 6 months, or ate most of their meals outside the home were excluded. The primary outcome was stroke, and the secondary outcomes were major adverse cardiovascular events and death from any cause.

Overall, 10,504 persons were randomized to the intervention group, and 10,491 participants were assigned to the control group. The mean age of the study participants was 65 years, with half of them being women. Seventy-three percent of study participants had a history of stroke, and 79% were using at least 1 antihypertensive medication. The mean 24-hour urinary sodium and potassium excretion were 4.3 g (187 mmol), and 1.4 g (36 mmol), respectively. At the end of the study, as measured in a subset of the sample, sodium excretion was decreased by 15.2 mmol (350 mg) and potassium excretion was increased by 20.6 mmol (803 mg) in the intervention group relative to the control group. This was accompanied by a BP reduction of −3.3/−0.7 mm Hg at the end of the study in the intervention group compared with the control group. A benefit with respect to the primary outcome was demonstrated in the intervention group, with a relative risk for stroke of 0.86 (95% confidence interval, 0.77-0.96)]. This benefit was consistent across all the prespecified subgroups. Major adverse cardiovascular events (relative risk, 0.87; 95% confidence interval, 0.80-0.94) and all-cause mortality (relative risk, 0.88; 95% confidence interval, 0.82-0.95) were also lower in the salt substitution group. In terms of adverse events, there was no difference in hyperkalemia across the groups, with a relative risk of 1.04 (95% confidence interval, 0.80-1.37).

## The Tweetchat

For the tweetchat, there were 206 active participants and 899 tweets. It was noted that salt has had an important role in society, from being the root of the words “salary” and “salad” and the phrases “salt of the earth” and “worth one’s salt” to being idioms referencing salt in a positive connotation in the common American English vernacular. Salt has been used as a means for food preservation and as a source of providing iodine supplementation. Participants reiterated that the source of sodium in diet (whether added in the household or in the food supply chain) varies greatly by region, which calls into question the external validity of the trial. Early in the discussion, it was noted that it is difficult to generalize the results of this trial to settings with lower levels of dietary salt than those in rural China, where the average daily sodium intake is >11,000 mg (480 mmol).^9^ The American Heart Association and the World Health Organization, among others, recommend limiting sodium intake to much lower levels of <2 g/d (87 mmol).^14^^,^^15^ However, many participants believed that the trial was indeed applicable to their patient population because many people with food insecurity rely upon inexpensive prepared meals with high sodium content.

**Figure 1:**
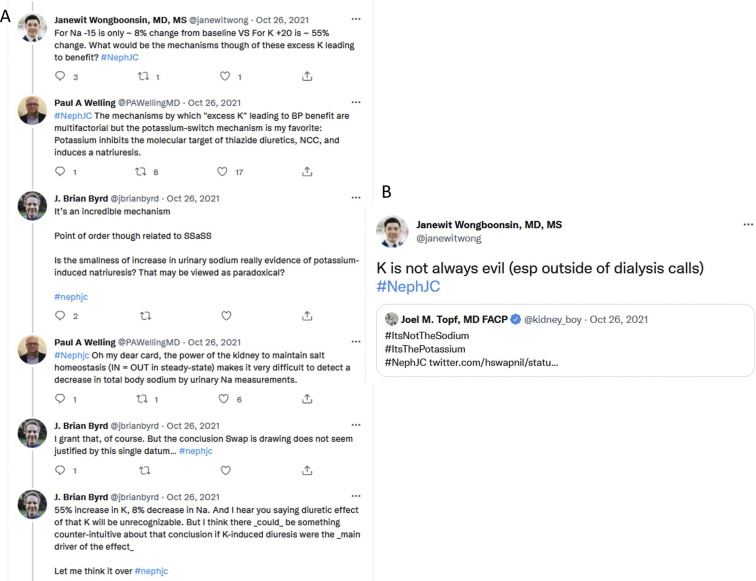
(A) Spirited discussion about the mechanism of effect of sodium or potassium given the small magnitude of change in sodium. (B) Need to embrace the benefit of potassium, which is otherwise a bad player in dialysis.

The magnitude of the actual average reduction of sodium intake at about 15 mmol/d from the baseline 187 mmol/d (about 8% decrease), was small, with a final intake of about 172 mmol/d or 4.0 g/d being noted. In contrast, the increase in potassium intake from a baseline of 36 mmol/d to 57 mmol/d (about 56% increase) provoked a longer discussion about the mechanism of benefit and whether it was mediated by sodium or potassium (Fig 1). In addition, since the decrease in BP seemed small (3.3/0.7 mm Hg), participants questioned whether this degree of BP reduction, even at a population-wide level, could completely explain the clinical benefits.

The incidence of hyperkalemia as reported in the study was not significantly different between the groups; however, that claim was taken with the proverbial grain of salt because of the lack of direct measurement of serum potassium and the exclusion of participants with serious kidney disease. Serious kidney disease was not clearly defined in the trial design, which brought into focus the issue of increased potassium intake exposure to the overall chronic kidney disease population in the face of universal implementation of this strategy, possibly in the food supply. It was noted that increased potassium intake does not always cause hyperkalemia because of the distal tubules’ kaliuretic abilities. A small trial has demonstrated the safety of a salt substitute, albeit in patients with stage 3 chronic kidney disease.^16^ Additionally, a modeling study of the impact of a potassium-enriched salt substitute in the general population sheds more light on this area.^17^ The risk assessment model in this study reported that despite hyperkalemia in chronic kidney disease causing an increase in mortality in this population, there would still be an overall reduction in net mortality because of a reduction in cardiovascular disease (11,000 more deaths from hyperkalemia but 21,000 fewer deaths from cardiovascular disease).^18^ Additionally, the model suggests an overall benefit of using a salt substitute in the entire population (450,000 fewer deaths annually).^17^

**Figure 2:**
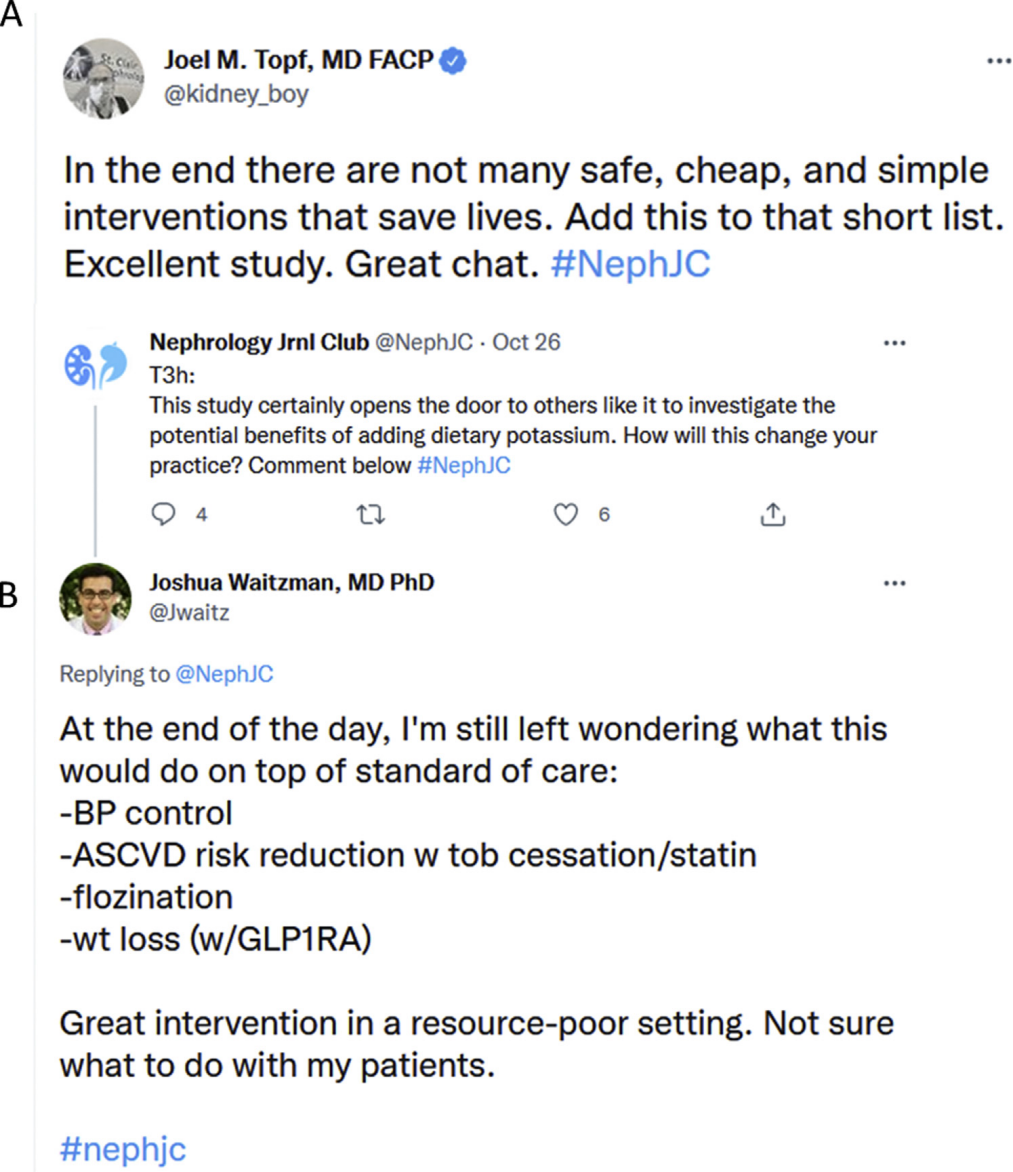
Two somewhat contrary takes on the same trial: (A) promising results that will save lives, and (B) underwhelmed about study utility and validity in the United States.

Although most participants believed that the results were encouraging, key questions remained with respect to the applicability of the intervention. In some countries (eg, India), salt substitutes are already available, and the change could be made quickly (Fig 2). In countries where the sodium is added in industrial food processing, change would require implementation at that level. There appears to be interest from the food industry, with the US Food and Drug Administration’s decision in 2020 to allow potassium chloride to be labeled as “potassium salt,” and research from the food industry showing that salt substitution in prepared foods can be achieved while maintaining taste.^17^^,^^19^^,^^20^ The clear, consistent benefit seen across all subgroups in the Salt Substitute and Stroke Study may spur action to implement change in our sodium and potassium intake without compromising on flavor.
